# Efficacy of the Self-management Support System DialBetesPlus for Diabetic Kidney Disease: Protocol for a Randomized Controlled Trial

**DOI:** 10.2196/31061

**Published:** 2021-08-17

**Authors:** Yuki Kawai, Akiko Sankoda, Kayo Waki, Kana Miyake, Aki Hayashi, Makiko Mieno, Hiromichi Wakui, Yuya Tsurutani, Jun Saito, Nobuhito Hirawa, Tadashi Yamakawa, Shiro Komiya, Akihiro Isogawa, Shinobu Satoh, Taichi Minami, Uru Osada, Tamio Iwamoto, Tatsuro Takano, Yasuo Terauchi, Kouichi Tamura, Toshimasa Yamauchi, Takashi Kadowaki, Masaomi Nangaku, Naoki Kashihara, Kazuhiko Ohe

**Affiliations:** 1 Department of Planning, Information and Management, University of Tokyo Hospital Tokyo Japan; 2 Department of Medical Science and Cardiorenal Medicine, Yokohama City University Graduate School of Medicine Yokohama Japan; 3 Department of Diabetes and Metabolic Diseases, Graduate School of Medicine, The University of Tokyo Tokyo Japan; 4 Department of Medical Informatics, Center for Information, Jichi Medical University Shimotsuke Japan; 5 Endocrinology and Diabetes Center, Yokohama Rosai Hospital Yokohama Japan; 6 Department of Nephrology and Hypertension, Yokohama City University Medical Center Yokohama Japan; 7 Department of Endocrinology and Diabetes, Yokohama City University Medical Center Yokohama Japan; 8 Division of Diabetes, Mitsui Memorial Hospital Tokyo Japan; 9 Department of Endocrinology and Metabolism, Chigasaki Municipal Hospital Chigasaki Japan; 10 Department of Diabetes and Endocrinology, Saiseikai Yokohamashi Nanbu Hospital Yokohama Japan; 11 Department of Nephrology and Hypertension, Saiseikai Yokohamashi Nanbu Hospital Yokohama Japan; 12 Department of Diabetes and Endocrinology, Fujisawa City Hospital Fujisawa Japan; 13 Department of Endocrinology and Metabolism, Yokohama City University Graduate School of Medicine Yokohama Japan; 14 Department of Prevention of Diabetes and Lifestyle-Related Diseases, Graduate School of Medicine, The University of Tokyo Tokyo Japan; 15 Toranomon Hospital Tokyo Japan; 16 Division of Nephrology and Endocrinology, Graduate School of Medicine, The University of Tokyo Tokyo Japan; 17 Department of Nephrology and Hypertension, Kawasaki Medical School Kurashiki Japan

**Keywords:** diabetic kidney disease, microalbuminuria, albuminuria, diabetes mellitus, self-management support system, mHealth, randomized controlled trial, diabetes, kidney, chronic disease, support, self-management, efficacy, protocol, therapy, intervention, self-care, behavior

## Abstract

**Background:**

Diabetic kidney disease (DKD) is one of the main complications of type 2 diabetes mellitus (T2DM). DKD is a known risk factor for end-stage renal disease, cardiovascular disease, and all-cause death. Effective intervention for early-stage DKD is vital to slowing down the progression of kidney disease and improve prognoses. Mobile health (mHealth) is reportedly effective in supporting patients’ self-care and improving glycemic control, but the impact of mHealth on DKD has yet to be shown.

**Objective:**

The purpose of this study is to evaluate the efficacy of standard therapy with the addition of a self-management support system, DialBetesPlus, in patients with DKD and microalbuminuria.

**Methods:**

This study is a prospective, randomized, open-label, multicenter clinical trial. The target population consists of 160 patients diagnosed with T2DM accompanied by microalbuminuria. We randomly assigned the patients to 2 groups—the intervention group using DialBetesPlus in addition to conventional therapy and the control group using conventional therapy alone. DialBetesPlus is a smartphone application that supports patients’ self-management of T2DM. The study period was 12 months, with a follow-up survey at 18 months. The primary outcome was a change in albuminuria levels at 12 months. Secondary outcomes included changes in physical parameters, blood test results (glycemic control, renal function, and lipid metabolism), lifestyle habits, self-management scores, medication therapy, and quality of life.

**Results:**

The study was approved in April 2018. We began recruiting patients in July 2018 and completed recruiting in August 2019. The final 18-month follow-up was conducted in March 2021. We recruited 159 patients and randomly allocated 70 into the intervention group and 61 into the control group, with 28 exclusions due to withdrawal of consent, refusal to continue, or ineligibility. The first results are expected to be available in 2021.

**Conclusions:**

This is the first randomized controlled trial assessing the efficacy of mHealth on early-stage DKD. We expect that albuminuria levels will decrease significantly in the intervention group due to improved glycemic control with ameliorated self-care behaviors.

**Trial Registration:**

UMIN-CTR UMIN000033261; https://upload.umin.ac.jp/cgi-open-bin/ctr/ctr_view.cgi?recptno=R000037924

**International Registered Report Identifier (IRRID):**

DERR1-10.2196/31061

## Introduction

The increase in type 2 diabetes mellitus (T2DM) worldwide is the primary cause of the increasing prevalence of end-stage renal disease (ESRD) [1]. The number of ESRD patients under renal replacement therapy is estimated to be more than 3 million worldwide and is projected to increase to more than 5 million people by 2030 [2]. Therefore, effective management of diabetic kidney disease (DKD) is essential, especially in the early stages. It has been demonstrated that albuminuria is one of the earliest detectible clinical manifestations of kidney disease and is a potent risk factor for ESRD and all-cause death [3,4]. Renin-angiotensin blockades are approved as a strategy to reduce urinary albumin excretion and are in widespread use [5,6]. Recently, glucagon-like peptide-1 analogs and sodium-glucose cotransporter-2 inhibitors have also been shown to slow the progression of DKD potentially [7,8]. Despite all these pharmacological interventions, the overall prevalence of DKD did not change significantly among US adults with diabetes from 1988 to 2014 [9]. Moreover, with 415 million patients with diabetes reported worldwide in 2015, the number is still increasing and is predicted to rise to 642 million by 2040 [10]. There is an increasingly urgent need for a more effective methodology to prevent the progression of DKD.

Modification of dietary and exercise habits is still one of the most fundamental therapeutic strategies. However, a sufficient support system for patients’ self-care has not been established. Whereas mobile health (mHealth) interventions supporting patients’ self-management have shown effectiveness in glycemic control of T2DM [11-14], the effects of mHealth on DKD have yet to be evaluated.

In 2015, over half of the people with diabetes worldwide lived in Southeast Asia or the Western Pacific Region. In Asia, it is estimated that the number of patients with diabetes will increase 1.8 times by 2040, and ESRD prevalence will rise sharply relative to other regions [2,10]. However, a population-based approach is also reported to decrease diabetes-related ESRD successfully [15,16]. mHealth might be a promising population-based approach, providing basic treatment and reliable information with easy accessibility at low cost.

We previously reported that the self-management support system DialBetics significantly improved glycemic control in T2DM patients, possibly due to improvement in diet and exercise [17,18]. We have since developed an updated system, DialBetesPlus, to investigate the effect of mHealth on DKD. In this study, we investigate the impact of DialBetesPlus on patients with DKD and microalbuminuria.

## Methods

### Study Design

**Figure 1:**
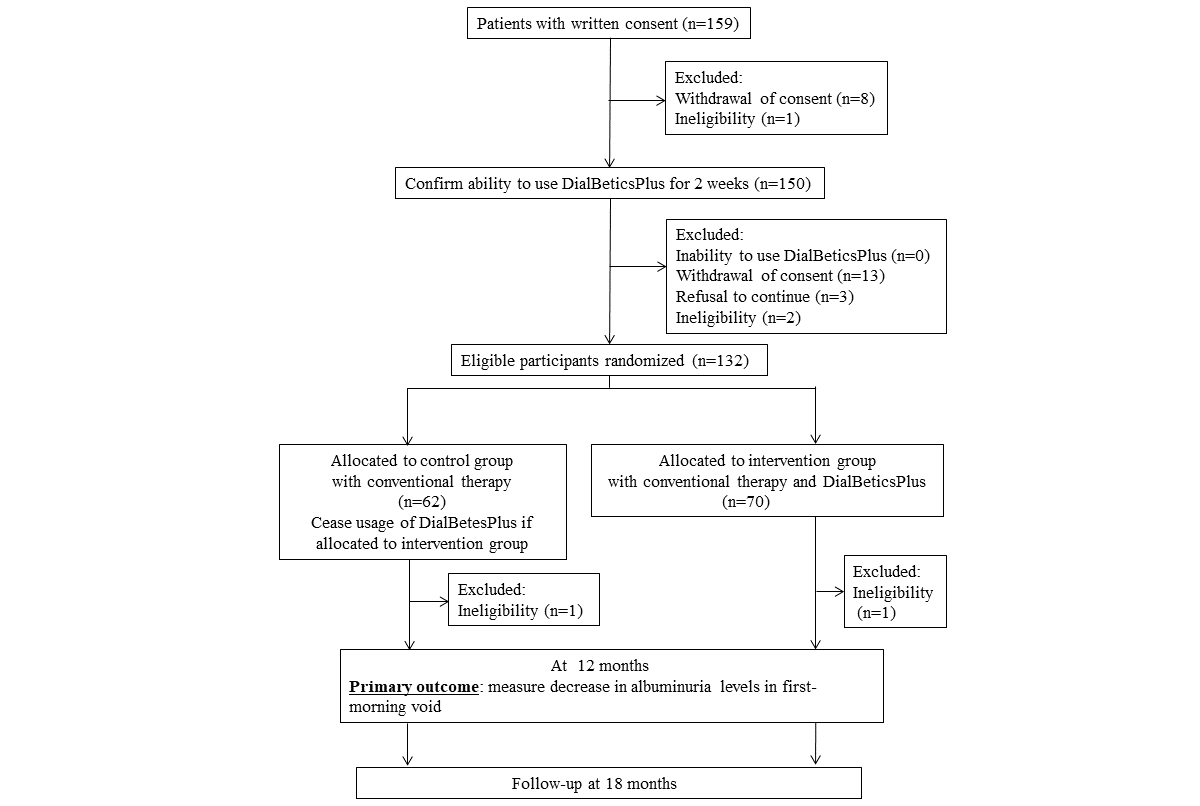
Protocol flowchart.

This study is a prospective, randomized, open-label, multicenter clinical trial. The study was conducted at 8 hospitals located in Tokyo and Kanagawa, Japan (Textbox 1). We aimed to evaluate the efficacy of DialBetesPlus on microalbuminuria in T2DM patients. Figure 1 shows a flowchart of this trial. The intervention group used DialBetesPlus and conventional therapy for 12 months, while the control group was treated with conventional therapy alone. The primary outcome was a change in albuminuria levels in a first-morning void after 12 months. The final follow-up was in 18 months, 6 months after the 12-month intervention period.

**List of trial institutions.**

- The University of Tokyo Hospital
- Yokohama City University Hospital
- Yokohama City University Medical Center
- Yokohama Rosai Hospital
- Saiseikai Yokohamashi Nanbu Hospital
- Fujisawa City Hospital
- Chigasaki Municipal Hospital
- Mitsui Memorial Hospital

### Participants

We recruited patients who passed all the inclusion and exclusion criteria before randomization (Textbox 2). The criteria were designed to include T2DM patients experiencing the early stage of DKD without any restrictions on physical activity. Eligible participants had T2DM with microalbuminuria (30-299 mg/g creatinine), hemoglobin A_1c_ (HbA_1c_) of 6.5% or more, and an estimated glomerular filtration rate (eGFR) of 45mL/min/1.73m^2^ or more. We obtained written, informed consent from all the patients participating in this study. Prior to the randomization, all enrolled patients were asked to use DialBetesPlus for 2 weeks to confirm their ability to use the system and devices properly. Eligible participants were those able to use DialBetesPlus for over 7 days during the initial 2-week confirmation period. The participants were randomized into either the intervention or control group in a one-to-one fashion based on albuminuria levels, gender, and age. The research team consisted of diabetologists, nephrologists, pharmacists, dieticians, nurses, a laboratory technician, and technical support experts. While the team handled technical troubles and monitored usage of the system, the patients continued to consult their attending doctors about their general health status. If 7 days passed without any input into the system, an alert was sent to encourage the patient to resume providing input. If 3 weeks passed without a patient inputting any data, we defined the patient as a dropout. Participants with whom the study team has lost contact were also treated as dropouts. The criteria for study discontinuance were a serious adverse event, patient request for discontinuance, pregnancy, or the judgment of a lead physician.

**Inclusion and exclusion criteria.**

**Inclusion criteria**

- Diagnosed with T2DM
- HbA_1c_ 6.5% or more
- Between 20 and 75 years of age
- BP lower than 180/110 mmHg
- eGFR 45mL/min/1.73m^2^ or more
- Two detected instances of microalbuminuria (30-299 mg/g creatinine) in spot urine samples prior to study enrollment
- BMI of 22 kg/m^2^ or more
- No history of severe hypoglycemia requiring additional medical support
- No history of the following symptoms indicating hypoglycemia within the last 3 months: palpitations, tremors, dizziness, anxiety, loss of consciousness, sweating, facial pallor, tachycardia, headache, sleepiness, blurred vision, or convulsions
- Regular patients of hospitals listed in Textbox 1
- Signatories of the informed consent form

**Exclusion criteria**

- Use of cardiac pacemaker
- Hyperthyroidism diagnosis, under medication other than thyroid hormone supplementation in the last year
- Medical instability or exercise restriction as ordered by a physician, with autoimmune, heart, liver, digestive, neurological, or respiratory disease
- Hb less than 10 g/ dL
- Albumin 3.0 g/dL or less
- eGFR less than 45mL/min/1.73m^2^
- Those with preproliferative diabetic retinopathy within one year of signing consent forms
- Inability to exercise
- Pregnancy, potential planned pregnancy, or lactating
- Participation in other clinical trials
- Under a diet that restricts protein
- Judged as ineligible by doctor’s discretion for other reasons

### Design of DialBetesPlus

**Figure 2:**
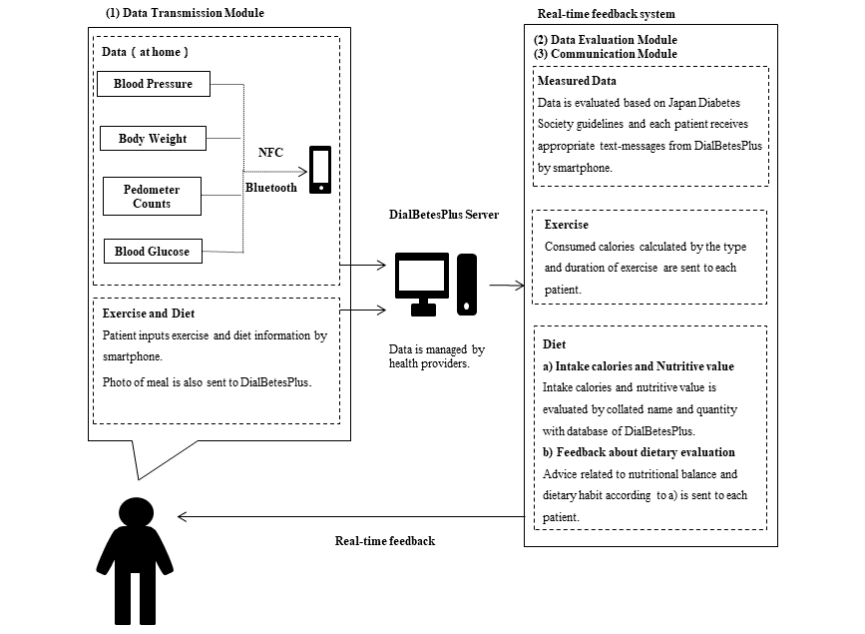
DialBetesPlus overview. NFC: near field communication.

The details of the DialBetesPlus system are shown in Figure 2. Patients measured blood glucose, blood pressure (BP), body weight, and pedometer counts at home. The data were transferred from each device to a smartphone by either Near Field Communication (NFC) or Bluetooth, and then immediately sent to a server, where they were automatically evaluated following the Japan Diabetes Society (JDS) guideline’s target values [19]. Optimal values include blood glucose below 110 mg/dl before breakfast and 140 mg/dl at bedtime, BP below 125/75 mmHg, and pedometer counts above 8000 steps per day. DialBetesPlus determined if each reading satisfies the JDS guideline requirements and immediately sent the results to the patient’s smartphone. Patients also entered the contents and quantity of their meals and the type and duration of exercise not counted by a pedometer. Then, their intake and consumed calories were automatically calculated and sent to the smartphone along with specific advice regarding lifestyle modifications based on JDS guidelines. Critical values with blood glucose levels above 400 mg/dl or below 70 mg/dl, systolic BP above 220 mmHg, or diastolic BP above 110mg were automatically reported to the research team, and the team informed attending doctors when necessary.

### The DialBetesPlus Intervention and Control

The patients allocated to the intervention group used DialBetesPlus for 12 months. Patients in the intervention group receive an NFC-enabled glucometer (MS-FR201B; Terumo), a Bluetooth-enabled BP monitor (HEM-7271T; Omron), a pedometer (MT-KT02DZ; Terumo), and a scale (HBF-255T; Omron). These devices were all paired with a single provided smartphone (Arrows F-02H; Fujitsu or Galaxy Note3 SC-01F; Samsung) that transmits readings to the DialBetesPlus server via a wireless network. In addition, as a part of standard therapy, the participants in the control group were provided with sphygmomanometers to measure BP at home.

In this study, we divided the participants into 2 models (a hospital-led model and a pharmacy-led model) depending on the location of study enrollment. In the hospital-led model (the University of Tokyo Hospital, Yokohama City University Hospital, Saiseikai Yokohamashi Nanbu Hospital, and Fujisawa City Hospital), the instructions included guidance on DialBetesPlus use provided by health care providers at the patient’s hospital. In a pharmacy-led model (run by the other 4 hospitals), instructions on using DialBetesPlus were provided by pharmacists supporting the study. The pharmacists are Nihon Chouzai Co, Ltd employees, with whom we signed a business cooperation contract to conduct the pharmacy-led model portion of the study.

### Sample Size

The primary outcome of this study is a change in albuminuria levels at 12 months. We estimated that baseline first-morning-void albuminuria level would be 200 plus or minus 200 mg/g creatinine and hypothesized the difference in the change in albuminuria between the intervention group and the control group at 12 months would be 100 plus or minus 200 mg/g creatinine. Based on previous reports [20,21], we calculated 63 patients were required in each group to achieve a 2-sided significance level of .05 and a statistical power of 80%. Factoring ineligibility and dropouts, we calculated a final target number of 80 patients per group.

### Study Outcomes

We defined the primary outcome as a change in albuminuria levels in the first-morning void over 12 months. A first-morning void is less influenced by hydration and physical activity than a spot urine sample [22-25]. As the participants in this study are restricted to patients with microalbuminuria but without macroalbuminuria, we validated albuminuria in a first-morning void.

The secondary outcomes were changes in physical parameters (BMI and BP), blood tests, lifestyle habits, self-management, medication therapy, adherence to the diabetes medication regimen, and quality of life (QoL). Blood test parameters included renal function (eGFR), glycemic control (HbA_1c_ and fasting blood glucose), and lipid metabolism (high-density lipoprotein cholesterol [HDL-C], low-density lipoprotein cholesterol [LDL], and triglycerides [TG]). We also assessed all-cause deaths, composite cardiovascular outcomes, composite renal endpoints, safety, and the usage frequency of DialBetesPlus.

Changes in lifestyle habits were assessed via food-log diaries covering 3 days. Dietitians calculated caloric intake and nutrient balance. The Summary of Diabetes Self-Care Activities Measure (SDSCA)—which evaluates 7 aspects of the diabetes regimen: general diet, specific diet, exercise, medication taking, blood-glucose testing, foot care, and smoking—is a widely used self-reporting tool for patients with diabetes in the United States [26]. We evaluated changes in self-management using the Japanese version of the SDSCA, the J-SDSCA. Change in medication therapy was assessed by evaluating prescriptions. Diabetes medication adherence was monitored using the 8-item Morisky Adherence Scale (MMAS-8). The MMAS-8 is reliable and valid in measuring the adherence of patients with multiple chronic conditions worldwide, including T2DM [27-29]. We measured patients’ QoL using the Japanese version of the Audit of Diabetes-Dependent Quality of Life (JP-ADDQoL). The ADDQoL is a diabetes-specific QoL measurement scale producing reliable and valid scores [30,31]. JP-ADDQoL also showed adequate reliability and acceptable validity [32].

Composite cardiovascular outcomes included the first occurrence and recurrence of myocardial infarction and stroke, the first occurrence of percutaneous coronary intervention and coronary artery bypass, hospitalization for unstable angina and heart failure, and cardiovascular death. The composite renal endpoint was defined as ESRD and more than a 2-fold increase in serum creatinine.

To ensure safety, we monitored the number of hypoglycemic events, other adverse events, and issues with the DialBetesPlus system throughout the study. The results of the hospital-led model and the pharmacy-led model will also be compared.

### Data Collection

At registration, we collected information on patients’ background, albuminuria level in a first-morning void, physical parameters, medications, blood tests, food-log diaries, J-SDSCA, MMAS-8, and JP-ADDQoL. Follow-up visits were scheduled at 2 months (plus or minus 4 weeks), 6 months (plus or minus 6 weeks), 12 months (plus or minus 6 weeks), and 18 months (plus or minus 6 weeks). We collected and recorded information according to the schedule in Table 1. Even if the interventional treatment was discontinued, we collected as much information as possible with the consent of participants.

**Table 1:** Assessment and follow-up schedule.

Assessments	Follow-up period				
	Registration	2 months	6 months	12 months	18 months
Patient background	●				
Albuminuria level in a first-morning void	●			●	●
Albuminuria level in a spot urine			●		
Physical parameters	●		●	●	●
Medication therapy	●	●	●	●	●
Blood test	●	●	●	●	●
Dietary intake log	●		●	●	●
J-SDSCA^a^	●		●	●	●
MMAS-8^b^	●			●	●
JP-ADDQoL^c^	●			●	
All-cause death		●	●	●	●
Composite cardiovascular outcome		●	●	●	●
Composite renal endpoint		●	●	●	●
DialBetesPlus safety questionnaire				●	
Safety		●	●	●	●
DialBetesPlus usage frequency			●	●	

^a^J-SDSCA: Japanese version of the Summary of Diabetes Self-Care Activities Measure.

^b^MMAS-8: 8-item Morisky Adherence Scale.

^c^JP-ADDQoL: Japanese version of the Audit of Diabetes-Dependent Quality of Life.

### COVID-19 Related Adjustments

Due to the COVID-19 pandemic, we made some adjustments to ensure complete data collection if participants canceled their hospital visits to avoid infection risk. The Research Ethics Committee of The University of Tokyo Graduate School of Medicine and its affiliated institutions formally approved adjustments to the trial protocol. First, we adopted a self-administered blood collection kit to measure blood glucose, HbA_1c_, eGFR, HDL-C, LDL-C, and TG levels when participants canceled in-person hospital visits. Albuminuria levels were also measured using the first-morning void mailed with the self-collected blood sample. We can conduct a blood examination by collecting 65 µl of blood from a fingertip with the self-administered blood collection kit, Ouchide-doc (Halmek Ventures, Inc). It is reported that the assay results with this blood collection method are quite comparable to the conventional methods used in hospitals [33]. Second, to minimize the face-to-face contact for data collection, we sent questionnaires to participants' homes in advance of their hospital visits.

### Ethics and Dissemination

The study is being carried out in compliance with the Declaration of Helsinki. This protocol and informed consent forms were approved by the Research Ethics Committee of The University of Tokyo Graduate School of Medicine and affiliated institutions. This study was registered in the University Hospital Medical Information Network Clinical Trials Registry (UMIN000033261) [34].

All participants are included after providing their signed and informed consent to participate in the trial. The participants are also informed of their right to withdraw from the study at any time. After the study concludes, data will be accessible by study groups for analysis and dissemination. All results of any analyses will be presented at major national and international scientific conferences and submitted for peer-reviewed journals of international repute and visibility.

### Statistical Analysis

Data regarding patients’ characteristics are presented as mean (SD) or median (IQR). We will compare changes in albuminuria levels, physical parameters, blood tests, and nutritional intake between the intervention and control groups. These will be analyzed using the 2-tailed *t* test or Mann-Whitney U test, as appropriate. Changes in J-SDSCA, MMAS-8, and JP-ADDQoL scores will also be analyzed using the Mann-Whitney U test. We will compare the proportion of hypoglycemia during the study in the intervention group to the proportion in the control group using Fisher's exact test. *P* values <.05 will be considered statistically significant. Statistical analyses will be performed using SAS (version 9.4; SAS Institute Inc).

## Results

The study was approved in April 2018. We started recruiting patients in July 2018 and completed recruitment in August 2019. The final 72-week follow-up was completed in April 2021. The first results are expected to be available later in 2021.

We recruited 159 participants with written informed consent (Figure 1). We had 24 participants excluded due to withdrawal of consent (21/159, 13%) and ineligibility (3/159, 2%). No participants were excluded due to an inability to use DialBetesPlus. A participant in the control group and another in the intervention group were also excluded after randomization due to the late discovery of ineligibility. The baseline characteristics of the remaining 133 patients are shown in Multimedia Appendix 1. Data for continuous variables are expressed as mean (SD) or median (IQR).

Of the 150 participants, 3 (2%) subsequently declined to continue and were excluded, resulting in 132 randomized participants. Table 2 displays the baseline demographic characteristics of the remaining 130 participants, excluding the 2 late-discovered ineligible participants. Data for continuous variables are expressed as mean (SD) or median (IQR). *P* values for continuous variables were calculated with 2-tailed Student’s *t* test or Mann-Whitney’s U test. *P* values for categorical variables were calculated with Fisher’s exact test.

The baseline characteristics showed no significant differences between the control group and the intervention group.

**Table 2:** Baseline patient characteristics.

Characteristics			Total (n=130)		Control (n=61)		Intervention (n=69)		*P* value	
Age (years), mean (SD)			59.5 (9.4)		60.5 (8.7)		58.7 (10.0)		.28	
**Sex, n (%**)										.55
	Male	96 (73.8)		47 (77.0)		49 (71.0)				
	Female	34 (26.2)		14 (23.0)		20 (29.0)				
**Physical parameters, mean (SD)**										
	BMI (kg/m^2^)	28.5 (4.6)		28.3 (4.0)		28.6 (5.2)		.67		
	Systolic BP^a^ (mmHg)	133.2 (16.7)		133.8 (17.2)		132.8 (16.3)		.73		
	Diastolic BP (mmHg)	82.0 (10.7)		83.2 (10.2)		80.9 (11.0)		.23		
**Smoking status, n (%)**										.79
	Nonsmoker	53 (40.8)		23 (37.7)		30 (43.5)				
	Current smoker	29 (22.3)		14 (23.0)		15 (21.7)				
	Ex-smoker	48 (36.9)		24 (39.3)		24 (34.8)				
Duration of diabetes (years), mean (SD)			13.1 (7.2)		12.5 (6.4)		13.7 (7.9)		.35	
**Laboratory test, median (Q1-Q3)**										
	Fasting plasma glucose (mg/dL)	144.0 (124.0-174.0)		139.0 (124.0-159.0)		150.0 (123.0-185.0)		.37		
	HbA_1c_^b^ (%)	7.5 (7.0-8.0)		7.4 (6.9-7.9)		7.5 (7.1-8.1)		.31		
	LDL^c^ cholesterol (mg/dL)	99.0 (78.0-117.0)		106.0 (78.0-117.0)		98.0 (83.0-112.0)		.55		
	HDL^d^ cholesterol (mg/dL)	49.5 (42.9-60.8)		49.0 (44.0-60.8)		50.0 (42.0-60.5)		.64		
	Triglycerides (mg/dl)	155.5 (100.0-261.0)		149.0 (100.0-261.0)		158.0 (100.0-252.0)		.64		
	Creatinine (mg/dL)	0.78 (0.65-0.92)		0.82 (0.68-0.93)		0.78 (0.62-0.92)		.33		
	eGFR^e^ (mL/min/1.73m^2^)	72.0 (61.8-85.3)		71.0 (63.8-83.0)		76.5 (59.5-85.5)		.29		
	UACR^f^ (mg/gCr)	36.4 (15.1-76.2)^g^		32.3 (14.8-70.6)		41.0 (15.1-78.0)		.49		

^a^BP: blood pressure.

^b^HbA_1c_: glycated hemoglobin.

^c^LDL-C: low-density lipoprotein cholesterol.

^d^HDL-C: high-density lipoprotein cholesterol.

^e^eGFR: estimated glomerular filtration rate.

^f^UACR: urine albumin-to-creatinine ratio.

^g^One case had a missing value.

## Discussion

The beneficial effect of mHealth on T2DM in improving glycemic control has been widely reported [14,35,36]. However, the impact of mHealth on DKD, one of the major microvascular complications of T2DM, has not yet been shown. To our knowledge, this is the first study evaluating the efficacy of mHealth on DKD in which microalbuminuria is the primary endpoint and eGFR is one of the secondary endpoints. Furthermore, because we followed participants for 6 months after the intervention, this study enables us to assess if the novel smartphone-based self-management support system DialBetesPlus can discernibly modify self-care behaviors in T2DM patients.

DialBetesPlus is an improved version of the previously reported DialBetics [17]. The main upgrade is providing feedback on a patient’s diet. The assessment is designed to provide positive feedback praising the patients’ achievement, encouraging patients, and improving their self-efficacy. The system assesses a patient’s diet precisely for each meal with an upgraded database. Additionally, patients can receive feedback on their daily and weekly diets to comprehensively look at their lifestyles.

While recent meta-analysis on mHealth shows that bidirectional communication between patients and health providers is indispensable for better glycemic control outcomes of T2DM patients [35,37], DialBetesPlus features a completely automated feedback system using the algorithm of DialBetics. Even though patients who used DialBetics cannot contact their health providers directly via DialBetics, a previous study showed significant reductions in HbA_1c_ (0.4% decrease), comparable to that achieved by other systems accompanied by interactive communication [17].

We hypothesize that albuminuria levels will significantly decrease in the intervention group compared to the control group due to improved self-care behaviors and glycemic control. This study may broaden the potential of mHealth to prevent the progression of T2DM microvascular complications.

## Appendix

Multimedia Appendix 1 Patient characteristics prior to randomization.

Multimedia Appendix 2 Physicians involved in participant recruitment.

## Abbreviations

BP: blood pressure
DKD: diabetic kidney disease
eGFR: estimated glomerular filtration rate
ESRD: end-stage renal disease
HbA_1c_: hemoglobin A_1c_
HDL-C: high-density lipoprotein cholesterol
JDS: Japan Diabetes Society
JP-ADDQoL: Japanese version of the Audit of Diabetes-Dependent Quality of Life
LDL-C: low-density lipoprotein cholesterol
mHealth: mobile health
MMAS-8: 8-item Morisky Adherence Scale
NFC: near field communication
QoL: quality of life
J-SDSCA: Japanese version of the Summary of Diabetes Self-Care Activities Measure
TG: triglycerides
T2DM: type 2 diabetes mellitus
