# Supplementary material for: Efficacy of the Self-management Support System DialBetesPlus for Diabetic Kidney Disease: Protocol for a Randomized Controlled Trial
Source: JMIR Res Protoc. 2021 Aug 17;10(8):e31061. doi: 10.2196/31061 (PMC8408755; doi:10.2196/31061)
Supplement: Multimedia Appendix 1 [file resprot_v10i8e31061_app1.docx]

| Characteristic | | Total participants (n=133) |
| --- | --- | --- |
| Age (years), mean (SD) | | 59.7 (9.4) |
| **Sex, n (%)** | |  |
|  | Male | 97 (72.9%) |
|  | Female | 36 (27.1%) |
| **Physical parameters, mean (SD)** | |  |
|  | BMI (kg/m^2^) | 28.4 (4.6) |
|  | Systolic BP^a^ (mmHg) | 133.2 (16.7) |
|  | Diastolic BP (mmHg) | 81.8 (10.7) |
| **Smoking status, n (%)** | |  |
|  | Nonsmoker | 55 (41.4%) |
|  | Current smoker | 29 (21.8%) |
|  | Ex-smoker | 49 (36.8%) |
| Duration of diabetes (years) | | 13.3 (7.6) |
| **Laboratory test, median (IQR)** | |  |
|  | Fasting plasma glucose (mg/dL) | 142.0 (123.0-172.0) |
|  | HbA1c^b^ (%) | 7.5 (7.0-8.0) |
|  | LDL^c^ cholesterol (mg/dL) | 98.0 (78.0-115.0) |
|  | HDL^d^ cholesterol (mg/dL) | 49.0 (42.5-60.8) |
|  | Triglycerides (mg/dl) | 155.0 (99.0-261.0) |
|  | Creatinine (mg/dL) | 0.8 (0.6-0.9) |
|  | eGFR^e^ (mL/min/1.73m^2^) | 72.0 (61.8-85.3) |
|  | UACR^f^ (mg/gCr)^*^ | 35.8 (15.1-75.3) |

**Multimedia Appendix 1.** Patient characteristics prior to randomization.

^a^BP: blood pressure.

^b^HbA1c: glycated hemoglobin.

^c^LDL-C: low-density lipoprotein cholesterol.

^d^HDL-C: high-density lipoprotein cholesterol.

^e^eGFR: estimated glomerular filtration rate.

^f^UACR: urine albumin-to-creatinine ratio.

*One case had a missing value.
