# Supplementary material for: Efficacy of the Self-management Support System DialBetesPlus for Diabetic Kidney Disease: Protocol for a Randomized Controlled Trial
Source: JMIR Res Protoc. 2021 Aug 17;10(8):e31061. doi: 10.2196/31061 (PMC8408755; doi:10.2196/31061)
Supplement: Multimedia Appendix 2 [file resprot_v10i8e31061_app2.docx]

**Multimedia Appendix 2.** Physicians involved in participant recruitment.

| The University of Tokyo Hospital | | | | |  |
| --- | --- | --- | --- | --- | --- |
|  | Naoko Arakawa | Yuta Hiraike | Yusuke Hirota | Hirofumi Honma |  |
|  | Jun Hosoe | Masahiko Iwamoto | Tomoya Kawaguchi | Yuka Kobayashi |  |
|  | Masaomi Miura | Sachiko Okazaki | Yoshitaka Sakurai | Takayoshi Sasako |  |
|  | Tomonobu Sawada | Minaka Takakura | Mikio Takanashi | Satoru Takase |  |
| ` | Masaki Tanaka | Gotaro Toda | Hironori Waki |  |  |
| Yokohama City University Hospital | | | | | |
|  | Ryoichi Akamatsu | Masanori Arai | Tatsuo Hashimoto | Rina Hiratsuka | |
|  | Takahiro Iijima | Ryota Inoue | Yuzuru Ito | Masayo Kimura | |
|  | Ryu Kobayashi | Rieko Kunishita | Mayu Kyohara | Hirotatsu Nakaguchi | |
|  | Naohito Okami | Tomoko Okuyama | Sumire Sunohara | Sakiko Terui | |
|  | Yu Togashi | Kazushi Uneda | Shingo Urate | Machiko Yabana | |
|  | Yoshihiko Yamada |  |  |  | |
| Yokohama Rosai Hospital | | | | | |
|  | Rei Hirose | Takuhei Hitsuwari | Hirofumi Horikoshi | Yoshitomo Hoshino | |
|  | Masahiro Ichikawa | Sho Katsuragawa | Haremaru Kubo | Takashi Sunouchi | |
|  | Tomoko Takiguchi | Takahiro Yamane |  |  | |
| Yokohama City University Medical Center | | | | | |
|  | Tatsuya Haze | Masahiro Ichikawa | Yoshinobu Kondo | Akeo Ohira | |
|  | Yasuyuki Sugiura |  |  |  | |
| Chigasaki Munisipal Hospital | | | | | |
|  | Masanori Hasebe | Taichi Suezono | Hitoshi Tamaki |  | |
| Saiseikai Yokohamashi Nanbu Hospital | | | | | |
|  | Akiko Kameda |  |  |  | |
